# Bruch’s membrane heparan sulfate retains lipoproteins in the early stages of age-related macular degeneration

**DOI:** 10.1073/pnas.2500727122

**Published:** 2025-06-13

**Authors:** Christopher B. Toomey, Savanna Pflugmacher, Kamalu Park, Jessica Pihl, Sammy Weiser Novak, Jessica Rodriguez, Maryam Jalali, Jaesoo Jung, Madeline Mozafari, Sima P. Omran, Cameron K. Pormir, Jill Hauer, Chelsea Painter, Evan Walker, Alex S. Huang, Daniela Boassa, James T. Handa, Teodor Aastrup, Philip L. S. M. Gordts, Jeffrey D. Esko

**Affiliations:** ^a^Viterbi Family Department of Ophthalmology and Shiley Eye Institute, University of California San Diego, La Jolla, CA 92093; ^b^Glycobiology Research and Training Center, University of California San Diego, La Jolla, CA 92093; ^c^Department of Cellular and Molecular Medicine, University of California San Diego, La Jolla, CA 92093; ^d^Waitt Advanced Biophotonic Core, Salk Institute, La Jolla, CA 92093; ^e^Department of Ophthalmology, Wilmer Eye Institute, Johns Hopkins Medical Institute, Baltimore, MD 21287; ^f^Attana AB, Stockholm, Sollentuna 191 62, Sweden; ^g^Department of Medicine, University of California San Diego, La Jolla, CA 92093

**Keywords:** age-related macular degeneration, lipoproteins, heparan sulfate, Bruch’s membrane

## Abstract

Lipoprotein retention in Bruch’s membrane is a key event in the pathobiology of early and intermediate age-related macular degeneration (AMD). However, the mechanism of lipoprotein retention in BrM is unknown. Given the established role of glycosaminoglycans (GAG) in binding lipoproteins, our laboratory sought to determine the role of GAGs in AMD BrM. In this study, BrM GAG content in AMD pathobiology was analyzed in human postmortem tissue. Strikingly, increased levels of highly sulfated heparan sulfate were present in AMD Bruch’s membrane as compared to non-AMD samples. In addition, using scanning electron microscopy of postmortem AMD tissue, we show aggregates of lipoprotein-like particles on the retinal pigmented epithelium side of Bruch’s membrane adjacent to heparan sulfate. We also show that heparin displaces lipoproteins rich in apolipoprotein A1 from human BrM, suggesting their identity as high-density lipoproteins. Using human BrM immobilized to quartz crystal microbalance biosensor (QCM) chips, we show that heparan sulfate is required for lipoprotein binding to BrM and soluble heparan sulfate can remove lipoproteins bound to BrM. Thus, our data establish that heparan sulfate regulates lipoprotein deposition in AMD BrM. These findings provide a foundation for targeted therapies capable of either preventing lipoprotein accumulation or removing drusen in the early and intermediate stages of AMD prior to vision loss.

## Significance

Lipoprotein-like particle deposition and aggregation in Bruch’s membrane is a key event in drusen biogenesis in the early and intermediate stages of age-related macular degeneration (AMD). Here, we establish that heparan sulfate, a sulfated extracellular glycosaminoglycan, is responsible for lipoprotein retention in the early and intermediate stages of AMD. These studies provide the foundation for the development of pharmaceuticals capable of cleansing Bruch’s membrane of drusenogenic lipoproteins in the early stages of AMD.

Age-related macular degeneration (AMD) is the leading cause of blindness in developed countries (1). This devastating disease affects 196 million individuals and is predicted to increase to 288 million by 2050 (1). Most of these patients suffer from the early and intermediate “dry” AMD and are currently without treatment options due to an incomplete mechanistic understanding of this complex disease. Genetic, biochemical, and histochemical analyses have converged to establish a role of extracellular matrix (ECM) metabolism in the biogenesis of drusen, a defining hallmark change in AMD (2–7). However, of the more than 100 clinical trials for AMD, none have targeted ECM changes. Treatment of AMD in early and intermediate stages has the potential to prevent vision loss early in the disease process and have an immense impact on quality of life (8) and health economics (9).

Drusen are the pathognomonic feature of early and intermediate AMD. Substantial clinical evidence indicates that drusen are not merely an association, but instead are causally related to the development and progression of AMD. Most notably, large drusen are associated with atrophy and disruption of the outer retinal layers, and diminished retinal sensitivity is observed directly overlying the drusen (10, 11). In addition, nascent geographical atrophy (GA), a degenerative process that damages the retina, occurs over large drusen (12, 13). The precise spatial relationship between drusen and damage observed in the outer retinal layers strongly suggests direct involvement of drusen in AMD progression. Additionally, histopathological examination of postmortem AMD eyes has consistently revealed drusen-related changes, such as disruption and dysmorphia of the RPE immediately overlying drusen (14, 15). It is noteworthy that drusen are characterized by high concentrations of oxidized lipoproteins and hydroxyapatite, which are believed to contribute to inflammation and serve as a focal point for the pathobiology of AMD (16, 17).

Drusen are extracellular aggregates of lipoprotein-like particles that form between the RPE basal lamina and the inner collagenous layer (ICL) of Bruch’s membrane (BrM). With normal aging, lipoproteins accumulate on top of the ICL of BrM prior to the onset of drusen (5). This finding highlights the early involvement of lipoprotein deposition in the pathogenesis of drusen. Basal linear deposits, which are histologically linked to AMD and drusen, have been characterized as lipoprotein-like deposits on top of the ICL of BrM (18). Drusen form on top of the ICL of BrM, exhibiting a composition of apolipoproteins and esterified cholesterol characteristic of plasma lipoproteins (5, 18). Taken together, these clinical studies suggest BrM lipoprotein accumulation and drusen formation drive AMD pathobiology.

BrM is an acellular 3-layered (inner collagenous, elastic, and outer collagenous layers) ECM abutted by the RPE and choriocapillaris endothelial cell basal lamina. BrM is composed of collagens (type IV collagen in RPE basal lamina and type I and III collagens in the ICL) (19–21), laminins (20, 22), fibronectin (21) and sulfated glycosaminoglycans (GAGs), including heparan sulfate (HS) (23–25). However, the compositional changes of BrM in AMD and the relationship of these changes to lipoprotein particle retention are not understood. Given the similarities between plasma and BrM lipoproteins, the sub-RPE lipoprotein retention hypothesis of drusenogenesis is analogous to the subendothelial ECM retention hypothesis in atherosclerosis (26, 27). Studies in atherosclerosis have shown that increased retention, rather than increased influx, of lipoproteins is the primary factor driving subendothelial retention (26, 27). This retention is facilitated by the interaction of negatively charged sulfated GAGs in the arterial ECM with binding sites on apolipoproteins made up of positively charged amino acids (28–31).

In this study, we sought to analyze the changes in GAG content and composition to BrM in AMD and to elucidate the role of BrM in lipoprotein retention. We show that the predominant GAG in BrM is HS, which is increased twofold in AMD macula compared to controls. In addition, we show the interaction of lipoproteins and sulfated GAGs by scanning SEM in AMD BrM and quantify the interaction between lipoproteins and human BrM HS using quartz crystal microbalance biosensor (QCM) containing immobilized BrM. This interaction can be disrupted with exogenous heparin, suggesting a promising therapeutic direction for drusen removal from the retina.

## Results

### Glycosaminoglycan Analysis of AMD BrM.

**Fig. 1.**
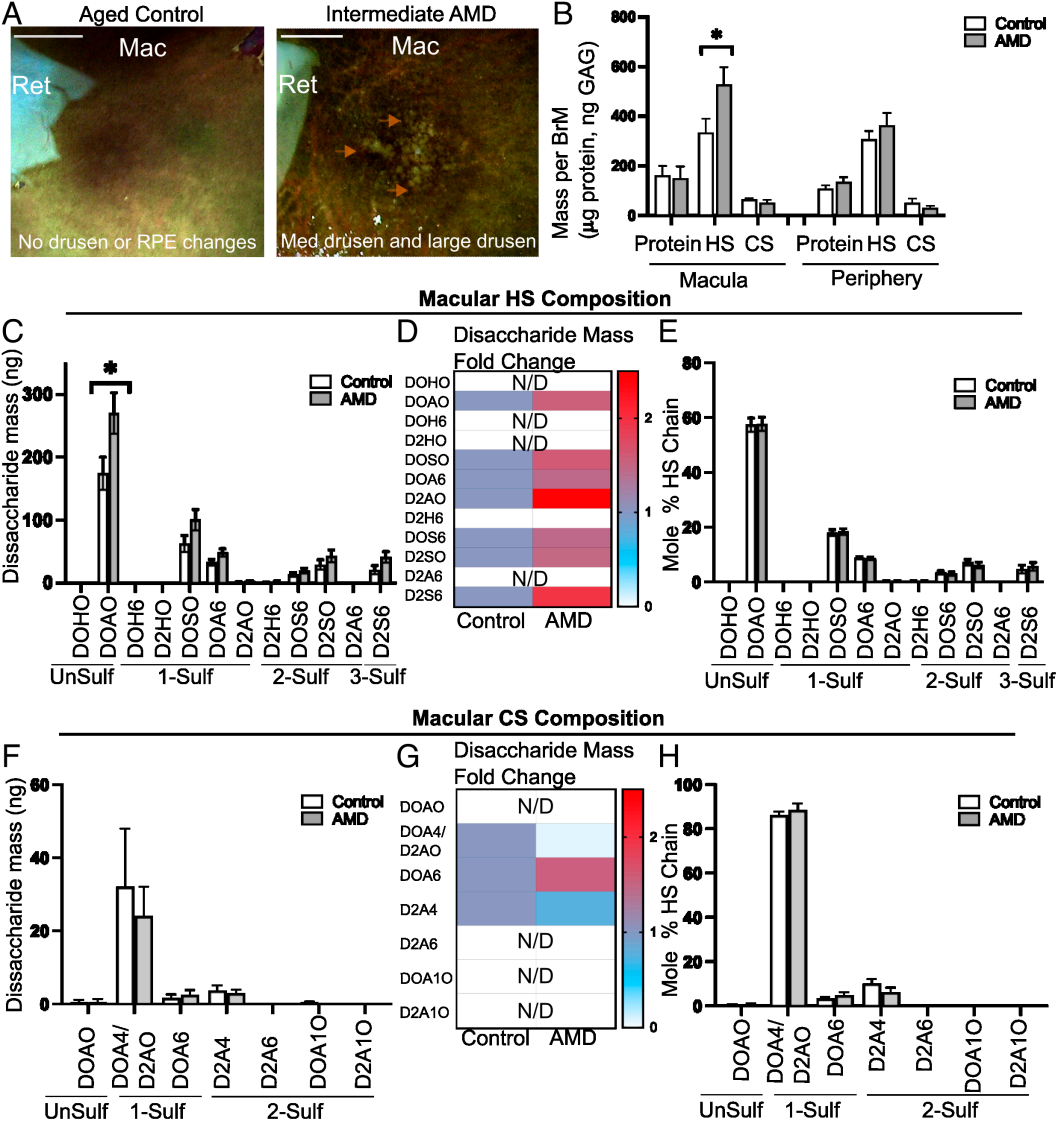
Glycosaminoglycan analysis of AMD macula BrM. Representative photos of postmortem globes after removal of the neural retina in normal aged controls (*Left*) and early/intermediate AMD (*Right*). Orange arrows indicate the presence of many intermediate and some large drusen in a patient with intermediate AMD (*A*, *Right* panel). (*B*) Analysis of the total BrM protein, heparan sulfate, and chondroitin sulfate content in BrM in the macula BrM and peripheral BrM shows that BrM contains a high content of HS, and we detected statistically significant higher level of BrM HS in the macula of patients with early and intermediate AMD (N = 7 subjects; 12 eyes, *SI Appendix*, Table S1 for demographics) compared to aged controls (N = 11 subjects; 17 eyes, *SI Appendix*, Table S1 for demographics). (*C*–*E*) Macular BrM HS disaccharide composition analysis shows a generalized increase HS disaccharide content including unSulf and highly sulfated species (*C* and *D*), but when normalizing to HS mole percentage all differences disappear (*E*) suggesting that the amount of HS and not the composition of HS is significantly altered in AMD BrM. In contrast, macula chondroitin sulfate composition is unchanged (*F*–*H*). Structures of disaccharide units used for glycosaminoglycan analysis are shown in *SI Appendix*, Fig. S1. Mac—macula, Ret—Retina, UnSulf—unsulfated, 1-Sulf—one sulfate group, 2-Sulf—two sulfate groups, 3-Sulf—three sulfate groups, N/D—not detected. * indicates *P* < 0.05. The scale bar indicates optic nerve head vertical diameter.

We analyzed the GAG composition of BrM in normal and AMD retina (Fig. 1 and *SI Appendix*, Table S1) from eyes genotyped for the major high-risk alleles (*SI Appendix*, Tables S2 and S3). Fundus photographs were taken of all postmortem globes to stage AMD. Representative fundus photos from an aged control (Fig. 1*A*, *Left*) and intermediate AMD with drusen deposition (Fig. 1*A*, *Right*) are shown. GAGs were extracted from human postmortem BrM tissue and quantified by glycan reductive isotope labeling (GRIL) LC/MS analysis of disaccharides liberated by digestion of the GAGs by bacterial chondroitinase ABC [to measure chondroitin sulfates (CS)] or heparin lyases [to measure heparan sulfate (HS)]. GRIL-LC/MS analysis of total GAG in macula and periphery of postmortem BrM tissue from aged controls and patients with early/intermediate AMD showed that HS is the predominant GAG present within BrM and the total amount of HS relative to protein is higher in early and intermediate AMD macular BrM (335 ± 56 ng HS controls vs 528 ± 70 ng HS AMD, *P* < 0.05, Fig. 1*B* and *SI Appendix*, Table S1). Disaccharide analysis of HS showed an increase in the unsulfated disaccharide D0A0 (174 ± 26 ng control vs 270 ± 32 ng AMD, *P* < 0.05), the monosulfated disaccharides D0S0 (63 ± 13 ng control vs 100 ± 16 ng AMD, *P* = 0.09) and D0A6 (32 ± 5 ng control vs 48 ± 6 ng AMD, *P* = 0.07), the disulfated disaccharide D2S0 (13 ± 8 ng control vs 19 ± 10 ng AMD, *P* = 0.25), and the trisulfated disaccharide D2S6 (20 ± 7 ng control vs 41 ± 9 ng AMD, *P* = 0.08) (Fig. 1 *C* and *D*, *SI Appendix*, Table S1). It is noteworthy that the relative mole percentage of the individual HS disaccharides did not change, suggesting a generalized increase in HS chains and no relative increase in specific disaccharides (Fig. 1*E*). The elevated amount of HS levels in BrM did not correlate with common AMD risk single nucleotide polymorphisms at Chromosome 1q32 or Chromosome 10q26 (*SI Appendix*, Tables S2 and S3). Interestingly, CS analysis showed no changes in composition or content in the AMD macula (Fig. 1 *F*–*H*). A comparison of GAGs obtained from the region peripheral to the macula in AMD and controls did not reveal any changes in composition or content of HS (*SI Appendix*, Fig. S2 *A*–C) or CS (*SI Appendix*, Fig. S2 *D*–F). Thus, the increase in HS was restricted to the region of high drusen content. The accumulation of highly negatively charged HS in AMD BrM implies that overall charge of BrM is dramatically increased.

Given the potential role of HS to trap lipoproteins in AMD BrM, we examined its localization in BrM and in drusen by immunohistochemistry. Samples were treated with heparin lyases, which depolymerizes the chain and leaves behind a core protein with HS “stubs” which contain a neoepitope recognized by mAb 3G10. Staining of treated samples showed that HS proteoglycans are present in AMD BrM and encapsulates drusen and/or are present in a thin layer of BlamD (Basal laminar deposits) overlying drusen (*SI Appendix*, Fig. S3, *Top* panel). Interestingly, staining samples with mAb 10E4, which specifically recognizes N-sulfated HS showed diminished N-sulfated HS underlying drusen (*SI Appendix*, Fig. S3, *Bottom* panel). It is also noteworthy that reduced HS staining was detected in the core of drusen (*SI Appendix*, Fig. S3, *Top* and *Middle* panels).

### Heparan Sulfate Colocalization With Lipoprotein Particles in AMD BrM.

**Fig. 2.**
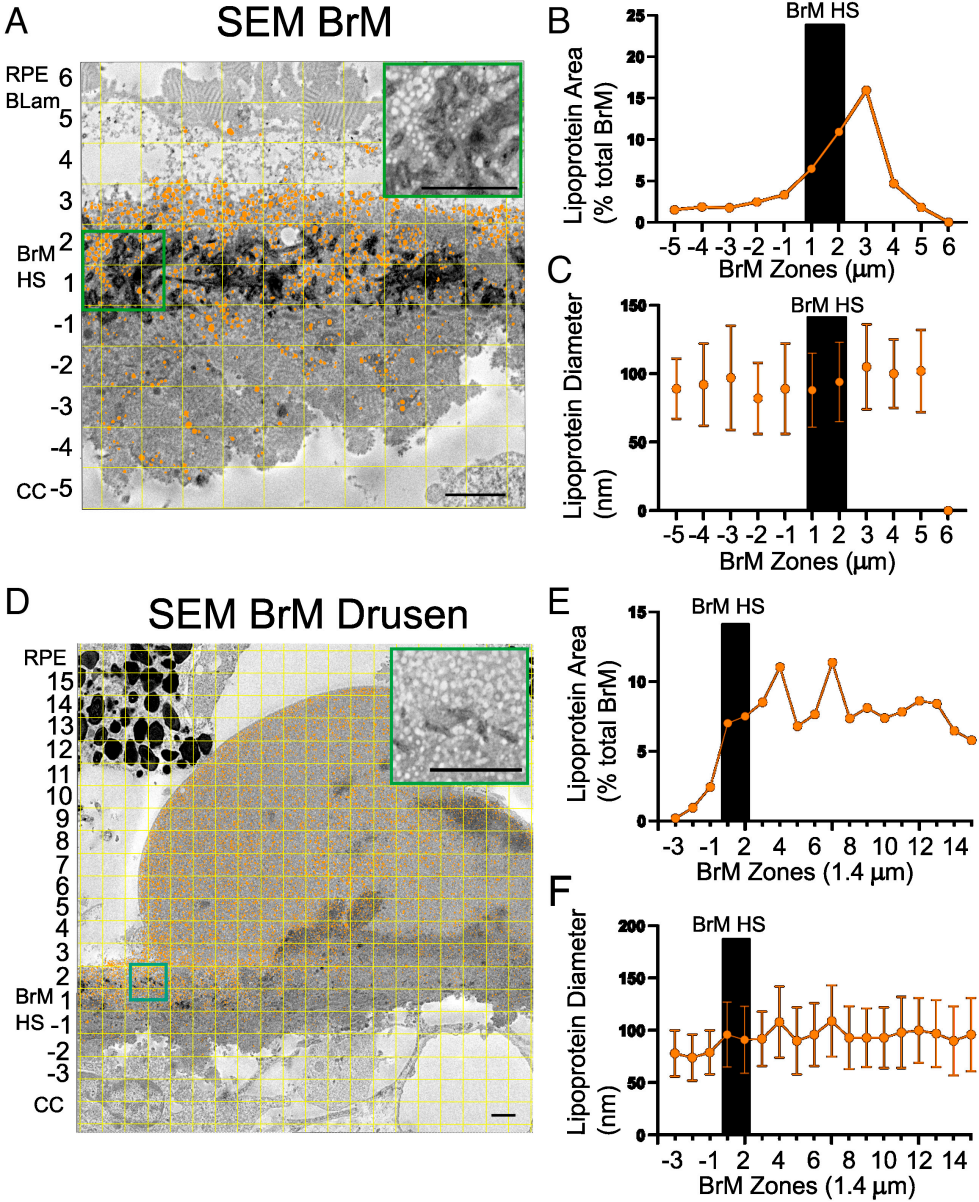
Particle retention anterior to HS in BrM. Representative scanning electron microscope sections stained with Ruthenium Red and subsequent particle analysis of BrM and drusen in 83-y-old female with early AMD (*A*-*F*) (Analysis was replicated in N = 3 regions from 2 donors, 83-y-old female with early AMD and 95-y-old female with early AMD). Particles were segmented based on morphology, and HS was identified by Ruthenium Red staining in BrM (*A* and *D*). 1 micron^2^ grid was created to analyze the spatial relationship between BrM HS and matrix vesicles. Zone −1 and Zone 1 are defined as the 1 µm^2^ posterior and anterior to the GAG staining in BrM (*A*), respectively. Each additional zone is 1 µm^2^ anterior or posterior. For analysis of drusen (*D*) 1.4 µm^2^ was used. Analysis of the coverage area (*B*) and diameter (*C*) of particles within BrM (orange and Zone −1 to 1 demarcates the most posterior border of HS staining in BrM) shows that the retention of particles occurs anterior to BrM HS (*B*), however, particle diameter is largely unchanged (*C*). This same analysis was performed underlying drusen (*D*–*F*), where particles also are present anterior to BrM HS (*D*–*E*) and particle diameter is also unchanged (*F*). RPE BLam—retinal pigmented epithelium basal lamina, BrM HS, Bruch’s membrane heparan sulfate, CC—choriocapillaris. (Scale bar, 1.4 µm, *A* and *C*.)

Scanning electron microscopy with Ruthenium Red staining was performed to examine the localization of HS in AMD BrM (N = 2 subjects) (Fig. 2*A*, black staining). Intense Ruthenium Red staining was observed in the ICL of BrM. Strikingly, spherical particles that resemble lipoproteins or vesicles within the ECM were abundant anterior to HS in BrM. We refer to these particles as lipoprotein-like particles given their morphology on SEM. The particles were manually annotated (orange) and their distribution was determined across the specimen by creating a 1 micron^2^ grid (Fig. 2*A*). Analysis of the coverage area of the particles (Fig. 2*B*) within BrM (orange and Zone −1 to 1 demarcates the most posterior border of HS staining in BrM) shows that the retention occurred anterior to BrM HS. The average diameter of these particles did not vary significantly (Fig. 2*C*). The particles that aggregate anterior to BrM HS in drusen (Fig. 2 *D*–*E*) had similar characteristics (Fig. 2*F*). These findings suggest that BrM HS might act as a nidus for lipoprotein retention.

### ApoA1-Containing Lipoprotein Particles are Eluted from BrM with Heparin.

**Fig. 3.**
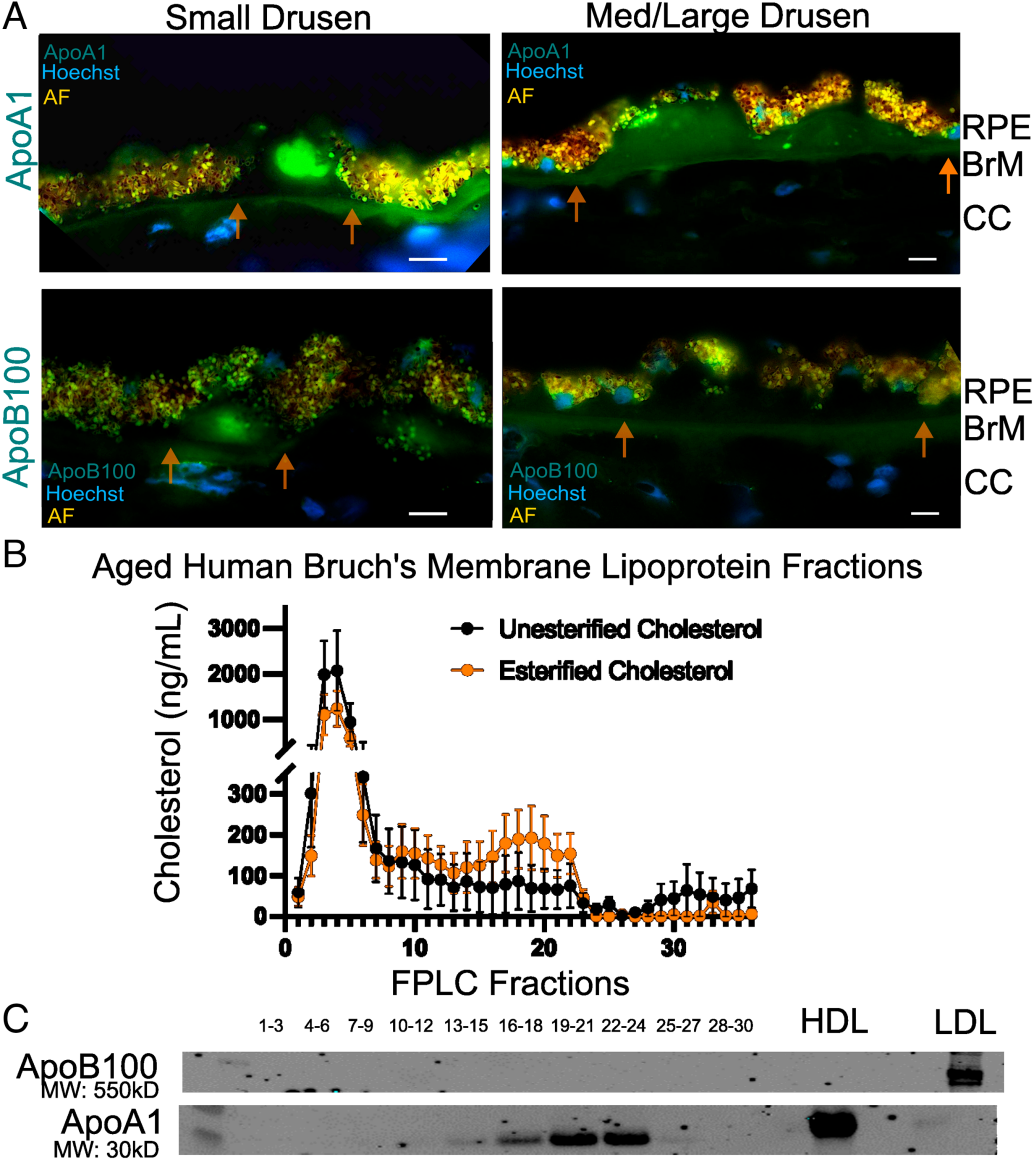
ApoA1-containing lipoproteins are removed from BrM with heparin. (*A*) Representative histology sections from a patient with intermediate AMD with small drusen (*Left*) and med/large drusen (*Right*) stained for lipoprotein markers ApoA1 (*A*, *Top* panel) and ApoB100 (*A*, *Bottom* panel). ApoA1 staining was observed frequently in drusen and Bruch’s membrane (N = 4 subjects, 73-y-old male with intermediate AMD, 86-y-old female without AMD, 90-y-old male with early AMD, and 95-y-old female with early AMD). (*B* and *C*) Bruch’s membrane from aged postmortem donors was minced and treated with heparin to elute lipoproteins (N = 12 subjects, 75 to 95 y-old). (*B*) FPLC fractionation shows two prominent peaks, a peak at fraction 4 largely composed of unesterified cholesterol (*B*, fraction 1 to 6), and no detectable levels of the apolipoprotein core proteins ApoA1 and ApoB100 were identified (*C*, fraction 1 to 6). A second peak at fraction 19 and 20 contains mostly esterified cholesterol (*B*, fraction 15 to 23) and contains the HDL-associated ApoA1 protein (*C*, fraction 16 to 24). HDL and LDL were positive controls for ApoA1 and ApoB100, respectively. AF—autofluorescence, FPLC—fast protein liquid chromatography, HDL—high-density lipoprotein. RPE—retinal pigmented epithelium, CC—choriocapillaris. (Scale bar, 10 µm.)

Based on the electron microscopy findings and prior work demonstrating the presence of lipoproteins in BrM, we examined the role of HS in lipoprotein retention (5). Immunohistochemistry of small and med/large drusen in patients with AMD had detectable levels of apolipoprotein ApoA1 [characteristic of high-density lipoproteins (HDL)] (Fig. 3*A*, *Top* panel). ApoB100 [characteristic of low density (LDL) and very low-density lipoproteins (VLDL)] was also present on some drusen but identified less frequently than ApoA1 by immunohistochemistry (Fig. 3*A*). To further analyze the lipoprotein class associated with BrM HS, BrM were isolated from aged donors (N = 12 subjects), gently minced, and incubated with 1 mg/mL of pharmaceutical grade unfractionated heparin to displace any bound lipoproteins. Displaced lipoprotein particles were separated by fast protein liquid chromatography (FPLC), and the fractions were analyzed for the presence of esterified cholesterol given the abundance of esterified cholesterol in all lipoprotein classes. FPLC fractionation showed two prominent peaks. A first peak, enriched for unesterified cholesterol (Fig. 3*B*, fraction 1 to 6), had no detectable levels of apolipoproteins ApoA1 (characteristic of HDL) and ApoB100 (characteristic of LDL and VLDL) (Fig. 3*C*, fraction 1 to 6). A second peak contained mostly esterified cholesterol (Fig. 3*B*, fraction 15 to 23) and HDL-associated ApoA1 (Fig. 3*C*, fraction 16 to 24). It is notable that ApoB100 particles were present in some samples but identified less frequently than ApoA1. These results indicated that HDL-like lipoproteins were present in BrM and dissociable by heparin, consistent with the idea that the particles were associated with endogenous BrM HS.

### Lipoprotein Binding in BrM Is Dependent on Heparan Sulfate.

**Fig. 4.**
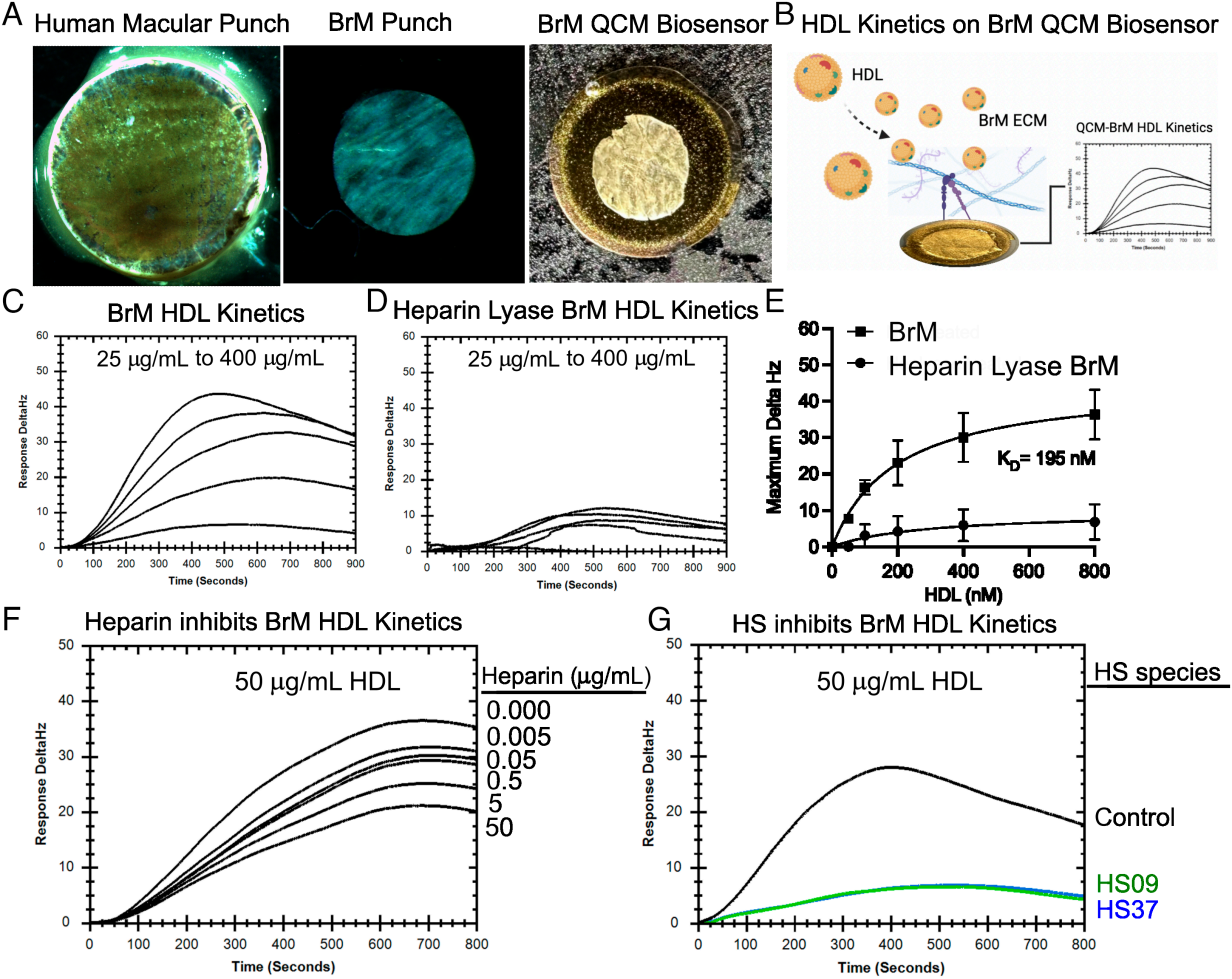
Lipoprotein particle binding to BrM is dependent on HS. Human BrM punch (N = 2 subjects, 86-y-old female and 80-y-old male, without AMD) were immobilized on gold-coated Attana QCM biosensors (*A*). Delta Hz frequencies represent changes in mass on the QCM biosensor. Human plasma HDL analytes were injected over BrM QCM biosensor to analyze binding affinity (*B*). At low flow rates (10 µL/s) HDL showed high affinity binding (K_D_ = 195 nM) (*C* and *E*). BrM treated with heparin lyases to remove endogenous HS treated in parallel failed to show binding (*D* and *E*). Soluble heparin diminished binding in a dose-dependent manner (*F*). Nonanticoagulant forms of heparin (HS09 and HS37) also blocked HDL binding to BrM compared to controls (*G*). QCM—quartz crystal microbalance, HDL—high-density lipoprotein.

To directly test whether lipoprotein retention depends on the physical interaction of the lipoproteins with BrM HS, we used a QCM. In these experiments, 4 mm diameter punch biopsies from human postmortem BrM (N = 2 subjects) were applied to gold plated QCM chips oriented with the ICL of BrM facing away from the chip and exposed to the analyte solution. The samples were air dried at 4 °C overnight and rehydrated in PBS. Human plasma HDL was used in these studies because the recovered lipoproteins from BrM is low. To measure binding, purified plasma HDL (25-400 µg/mL) was added as the analyte and flowed over to the chip at 10 µl/min (Fig. 4). Association times were measured for 900 s. Under these conditions, exogenous plasma HDL showed high affinity binding (~195 nM) to BrM and saturability (Fig. 4*C*). Binding was dependent on BrM HS because degradation of HS by heparin lyases dramatically reduced HDL binding (Fig. 4 *D*−*E*).

To test whether HDL binding to BrM could be blocked by exogenous GAG, we mixed heparin with plasma HDL and applied the mixture to the chip. Heparin showed a dose-dependent inhibition of binding (Fig. 4*F*). The unfractionated heparin used in this study binds and activates antithrombin which inhibits coagulation through inactivation of several plasma serine proteases. To circumvent this potentially significant side effect profile, we examined heparin-like material derived from MST mastocytoma cell lines (TEGA Therapeutics, Inc.) which lack anticoagulant activity (32). HS09, a form of nonanticoagulant MST heparin that lacks the key 3-*O*-sulfate group required for antithrombin binding, blocked HDL binding. HS37, which lacks 2-*O*- and 3-*O*-sulfation had a similar effect (Fig. 4*G*). HS37 does not bind to platelet factor 4 and thus has diminished ability to induce, another side effect of heparin, heparin-induced thrombocytopenia (32). These findings indicate that HDL binding can be diminished with modified forms of heparin, consistent with the observation that binding occurs to HS in BrM.

## Discussion

Prior research has established that lipoprotein aggregation in BrM is a prominent aging effect and a key early event in the formation of drusen and the pathogenesis of AMD. Our study adds to these findings by establishing that HS is significantly increased in AMD BrM and is a major factor in the retention of lipoprotein-like particles in the early and intermediate stages of AMD. Our findings also establish alteration of BrM HS is a viable pharmacologic target in AMD. The inhibition of lipoprotein binding to BrM by heparinoids suggests a potential pharmacological approach for preventing lipoprotein deposition. Their ability to displace lipoproteins bound to BrM suggests a potential pharmacological approach for reversing lipoprotein deposition.

These findings open additional unanswered questions regarding the regulation HS and origin and identity of the lipoprotein particles aggregating into BrM. HS is assembled by the copolymerization of glucuronic acid and N-acetylglucosamine residues to a linkage tetrasaccharide covalently bound to a proteoglycan core protein (33). A family of sulfotransferases and an epimerase modifies these residues in segments, creating sulfated domains of variable length interspersed by nonsulfated domains (33). The diversity of HS is due to variable chain length (catalyzed by the glycosyltransferase enzymes Ext1 and Ext2) and variable sulfation (catalyzed by the sulfotransferase enzymes NDSTs:N-acetylglucosamine N-deacetylation-N-sulfation, HS2STs:uronyl 2-*O*-sulfation, and HS6STs:N-acetylglucosamine 6-*O*-sulfation) (33). HS chains also undergo remodeling by heparanases, which trims the chains, and endosulfatases that selectively remove 6-*O*-sulfate groups after their presentation in the ECM (33). The factors that regulate the composition of the chains are diverse and reflect both the metabolic state of the cells, as well as the expression levels of the enzymes, and the core proteins. Nothing is known about the mechanism of HS regulation in BrM in healthy or AMD retina.

Histologically, prior to AMD onset, lipoprotein-like particles accumulate in BrM (18, 34). These particles appear to be heterogeneous in morphology and may be a mixture of membrane vesicles and lipoproteins like those found in plasma HDL. However, the origin and the subclass of the lipoprotein-like particles that aggregate in BrM are not well described. Prior studies have provided conflicting evidence, with some studies suggesting ApoB100 containing VLDL-like particle aggregates in BrM (35, 36), whereas recent evidence suggests ApoA1-containing HDL-like particle aggregates in BrM (25). Human RPE cells express apolipoproteins and other genes involved in lipoprotein metabolism (37). Notably, large population-based clinical studies have correlated high serum HDL levels and single nucleotide polymorphisms in the reverse cholesterol transport pathway to AMD (2, 38–41). These observations are increasingly relevant to the aging community, given the interest in the reverse cholesterol transport pathway and HDL serum profiles for the treatment of cardiovascular disease. In fact, in 2022, Nordestgaard and colleagues reported that cholesteryl ester transferase (CETP) deficiency, mimicking pharmacological inhibition of CETP, was associated with a lower cardiovascular morbidity but markedly higher risk of AMD (39, 42–44). In our analysis of BrM lipoprotein accumulation, we found both ApoA1 and ApoB100 containing lipoprotein particles were present in BrM and drusen, but ApoA1-containing lipoprotein particles appear to be the predominant lipoprotein particle based on our methodology. However, the pathobiology of HS retention extends beyond HDL particles in BrM, as HS has been shown to interact with a diverse range of macromolecules, including other ApoE containing lipoproteins, proteins, and extracellular vesicles such as exosomes (33, 45). Given that drusen formation occurs outside of the blood–retinal barrier, efforts to systemic lipoproteins could also affect AMD progression. Thus, determining the origin and precise lipoprotein profile in AMD is an important goal for future of aging research.

In atherosclerosis, it is noteworthy that once retained in the ECM, lipoproteins aggregate and undergo modifications including lipid oxidation which serve as a nidus for inflammation (46). Analogous biology appears to be occurring in BrM in patients with AMD (16, 17). A significant contrast lies in AMD, where the lipoprotein-like material remains extracellular due to the lack of immune cell infiltration, whereas atherosclerotic plaques typically exhibit lipid-laden macrophages. Thus, the role of BrM in sequestration of the extracellular deposits is an area of future study and relevant to AMD pathogenesis.

The application of QCM technology to AMD research is unique. The ability to immobilize BrM on gold chips allowed us to examine lipoprotein binding and its dependence on HS. Moreover, the method allowed us to show that exogenous heparins can block binding of lipoproteins to BrM. This observation supports work showing that heparin inhibits lipoprotein particles from binding to decellularized RPE cultures (25). The QCM technology demonstrated here is adaptable to test the binding properties of BrM with a range of analytes. Taken together, these observations open the possibility of using nonanticoagulant forms of heparin and possibly HS as agents to reduce further lipoprotein deposition in patients with AMD and possibly to remove drusen.

## Methods

### Glycan Reductive Isotope Labeling and Liquid Chromatography/Mass spectrometry of GAG.

Donor eyes were procured from the San Diego Eye Bank from 65 to 90 y-old, including patients with AMD. A <12-h death-enucleation interval and <24-h receiving interval were used to ensure GAG stability. After removal of the neurosensory retina, high-resolution, digital color fundus photographs were taken of the posterior pole. Analysis of subretinal drusenoid deposits was not possible in postmortem specimens. AMD grading was performed according to the 9-step Minnesota Grading scale and AREDS categories (47). Macular and superior mid-peripheral punches (6 mm diameter) of the RPE/BrM/choroid complex were performed. BrM was isolated from the RPE/BrM/choroid complex by microscopic dissection. Clinical data including past ocular history and AMD status were documented. PCR genotyping was performed on ocular tissue for the common *CFH* (rs1061170) and *HTRA1/ARMS2* (rs11200638) single nucleotide polymorphisms (*SI Appendix*, Tables S2 and S3). GAGs were quantified by GRIL-LC/MS (48). Briefly, GAGs were isolated from tissue after protease digestion and DEAE anion-exchange chromatography, and the reducing end of lyase-generated disaccharides were tagged with [^12^C_6_]aniline. Samples were mixed with [^13^C_6_]aniline-tagged GAG disaccharides standards and quantified by LC/MS. Individual disaccharides were quantified relative to known amounts of mass-tagged standards and summed to give the total weight of GAG and disaccharide in nanograms.

### Human CFH and ARMS2/HTRA1 Genotyping.

Postmortem human tissue was used in the Qiagen DNeasy Blood and Tissue Kit (Cat. No. 69504) spin column procedure and followed with a PCR using a 55 °C annealing temperature.

HTRA1 Forward Primer: CGGATGCACCAAAGATTCTCC; HTRA1 Reverse Primer: TTCGCGTCCTTCAAACTAATGG; CFH Forward Primer: AATCACAGGAGAAATAAATATAGG; CFH Reverse Primer: ATGTAACTGTGGTCTGCGCTT. Following PCR amplification, samples were submitted to Azenta Life Sciences for Sanger Sequencing. Results from the sequencing were compared using the Benchling platform. The DNA sequence of each gene was added to the program from the ensembl ID; CFH (ENST00000367429), HTRA1 (ENST00000368984). The sequencing from each patient was aligned with the ensembl ID sequence to check for differences at the SNP sites.

### GAGomics Data Analysis.

Subject and eye-level demographic and clinical characteristics are displayed as count (%) and mean (95% CI) for categorical and continuous variables, respectively. Comparisons were made across AMD status, HTRA1 Status Genotypes, and CFH Status Genotypes. Subject-level continuous and categorical parameters were compared using t-tests and Fisher’s Exact tests, respectively. Eye-level continuous parameters were compared using linear mixed-effects models. All linear mixed-effects models were fit with a random intercept to adjust for within-subject variability, controlling for the correlated measurements of subjects with both eyes included in the study. When comparisons were made across three subgroups, comparisons of subject-level continuous parameters were evaluated using ANOVA, and comparisons of eye-level continuous parameters were evaluated using mixed-design ANOVA. The statistical analysis was conducted using the R programming language for statistical computing, Version 4.4.0 [R Core Team (2022). R Foundation. Vienna, Austria]. *P*-values less than 0.05 were considered statistically significant.

### Heparan Sulfate Immunohistochemistry.

Postmortem human tissue isolated from an 86-y-old female without AMD and a 90-y-old male with early AMD from the San Diego Eye Bank with a postmortem interval of 16 h. The posterior pole was isolated, and a 4 mm punch of Retina/RPE/Choroid was obtained from the macula and embedded in OCT and flash frozen without fixation. Cross-sections (10 µm) were cut with assistance from the La Jolla Institute for Immunology Histology Core. Frozen sections were air dried, fixed with 95% ethanol, and treated with 0.3% hydrogen peroxide in PBS, and then blocked with 3% BSA in PBST. Heparin lyase digestion was performed with Hep I/II/III 5 mU/mL for 1 h at room temperature as a control. The tissue was then treated with primary antibody 10E4 or 3G10 to stain for N-sulfated HS chains or HS stubs (49). Slides were washed and then treated with secondary donkey anti-mouse IgM peroxidase and anti-mouse IgG peroxidase, respectively, at 1:2,000. 3,3′-diaminobenzidine and hematoxylin treatment were performed and then slides were imaged on a Zeiss AxioLab Microscope at 100× magnification.

### Scanning Electron Microscopy Analysis of GAGs in Human BrM.

Postmortem human tissue isolated from N = 2 subjects (83-y-old female with early AMD and 95-y-old female with early AMD) from the San Diego Eye Bank with a postmortem interval of less than 5 h. The globe was immediately preserved in 4% paraformaldehyde. The posterior pole was isolated, and a 2 mm punch of Retina/RPE/Choroid was obtained from the macula and fixed in 2.5% glutaraldehyde, 4% paraformaldehyde in 100 mM sodium cacodylate buffer. Samples were rinsed and stored in a cryoprotectant 4:3:3;1 × PBS:glycerol:ethylene glycol. A foveal sample was bisected and embedded in agarose, and 50 to 200 µm vibratome sections were cut. The most intact sections were then used for staining and fixation with dehydration in ascending concentrations of ethanol and embed in epoxy resin. The samples were then postfixed with 1.2% glutaraldehyde with 0.05% ruthenium red and 0.06 M sodium cacodylate buffer, then further postfixed with 1.6% osmium with 0.05% ruthenium red 0.06 M sodium cacodylate buffer (50). Ruthenium red is a cationic dye used to stain negatively charged GAGs. Ultrathin sections (60 to 80 nm) were then cut using a Leica UC7/FC7 ultramicrotome (Waitt Advanced Biophotonics Core, Salk Institute). SEM imaging was performed on the Carl Zeiss SIGMA Variable Pressure Field Emission Gun Scanning Electron Microscope equipped with an ATLAS montage imaging module and the Shuttle and Find correlative microscopy navigation module to generate images and high-throughput workflow (Waitt Advanced Biophotonics Core, Salk Institute). Lipoprotein-like particles were identified based on the characteristic spheroid shape and homogenous electron lucent core morphology. SEM sections with drusen deposits (N = 3 sections) in BrM were analyzed using segmentation with ImageJ to determine the distribution of particle diameter and percent area of occupied by particles in various zones. Ruthenium red staining was used to identify GAGs on SEM images. Zones (1 µm^2^) were created for analysis of GAG-lipoprotein spatial relationship where the border between Zone −1 and Zone 1 is defined as the 1 µm^2^ posterior and anterior to the GAG staining in BrM, respectively. Each additional zone is 1 µm^2^ anterior or posterior. For analysis of drusen, 1.4 µm^2^ was used. Statistical analysis of particle size and spatial relationship between GAGs was performed using GraphPad software. Representative images were displayed.

### ApoA1 and ApoB100 Immunohistochemistry.

Postmortem human tissue isolated from 4 subjects (73-y-old male with intermediate AMD, 86-y-old female without AMD, 90-y-old male with early AMD, and 95-y-old female with early AMD) from the San Diego Eye Bank with a postmortem interval of less than 24 h. Briefly, macular sections were embedded in OCT and flash frozen, and thin sections were cut with assistance from the La Jolla Institute for Immunology Histology Core. Frozen sections were dried, fixed with 95% ethanol and treated with 0.3% hydrogen peroxide in PBS and then blocked with 3% BSA in PBST. The tissue was treated with primary antibody Anti-Human ApoA1, rabbit IgG (R7D Systems Cat# MAB36641). For ApoB100, the primary antibody used was Biotin-Goat anti-Human ApoB100 (Academy Bio-Medical Company, Cat# 20B-G1a). Slides were washed and treated with 1:2000 dilution secondary antibody. For the ApoA1 slides, the secondary antibody used was Invitrogen Alexa Fluor 488 donkey anti-rabbit IgG (Invitrogen, ThermoFisher Cat# A21206), and for the ApoB100, the secondary antibody was Streptavidin Alexa Fluor 488, conjugate (Invitrogen, ThermoFisher, Cat# S32354). RPE autofluorescence was distinguished using unstained slides and overlay with 596 excitation and 615 emissions. Control slides did not have secondary antibody. Representative images were displayed. Nuclear staining done using 1:5000 Hoechst stain. Slides were imaged on a Zeiss AXIO Observer D1 Microscope at 63x magnification.

### BrM Lipoprotein Particle Separation by Fast Protein Liquid Chromatography.

Aged BrM (N = 12 subjects, aged 75 to 95 y-old) was isolated using gentle mechanical dissection and then minced with a razor blade and then incubated overnight at 4 °C with 1 mg/mL pharmaceutical grade heparin (derived from porcine intestinal mucosa, Shenzhen HepaLink Pharmaceuticals) in sterile PBS on a plate rocker. Soluble particles were then concentrated to 250 µL in volume using 3000 molecular weight cutoff centrifugal filters (Amicon Ultra-3 K, Sigma-Aldrich) and separated by gel-filtration fast protein liquid chromatography. Samples were loaded on a GE Superose 6 10/30 GL column in 0.15 M sodium chloride containing 1 mM ethylenediaminetetraacetic acid and 0.02% sodium azide, pH 7.4. Thirty six fractions of 0.5 mL were collected (0.5 mL/min). Esterified and unesterified cholesterol were determined using Amplex Red Cholesterol Assay Kit on FPLC fractions (Invitrogen; A12216).

### Western Blots.

FPLC samples were concentrated (Amicon Ultra-3 K, Sigma-Aldrich), and then pooled fractions were reduced with beta-mercaptoethanol, fractionated by SDS-PAGE on 4-12% Bis-Tris gradient gels (NuPage, Invitrogen), and transferred to a PVDF membrane (Power Blotter Select Transfer Stack; Thermo Fisher Scientific; PB5210) using lab standard methods. Membranes were blocked with fish serum blocking buffer (Thermo Scientific; 37527) for 1 h and incubated overnight at 4 °C with respective antibodies. Secondary antibodies were incubated for 1 h the following day and visualized with an Odyssey IR Imaging System (LI-COR Biosciences). Western Blot antibodies include Biotin-Goat Anti-Human ApoB-100 (Academy Biomedical Company, Inc; 20B-Gla; 1:5000), rabbit monoclonal anti-hApoA1 (R&D Systems; MAB36641; 1:500), Streptavidin (LicorBio; 926-68031; 1:5,000), and goat anti-rabbit (LicorBio; 926-32211; 1:5,000)

### Quartz Crystal Microbalance of BrM using Heparin and Full-length HS species.

BrM was isolated using gentle mechanical dissection of the overlying RPE and underlying choroid with Finesse Maxgrip forceps and a Finesse Flexloop (Alcon, CA) with a dissecting microscope, and gentle decellularization was performed with hypotonic solutions. Four-mm diameter tissue biopsy punch (Miltex, NJ) of isolated BrM was positioned on gold-coated Attana QCM chips and dried at 4 °C overnight (Attana Life Science and Diagnostics, Sweden). BrM punches were treated with a mixture of heparin lyases I/II/III (2.5 mU of each/mL, overnight at room temperature). The QCM-BrM chips and QCM-BrM chips treated with heparin lyases were analyzed in parallel on the Attana QCM biosensor to examine the binding of lipoproteins to BrM. After overnight stabilization, 25 to 400 µg/mL lipoprotein analyte in PBS was injected at 10 µL/min at 22 °C. Association and dissociation were monitored for 500 s and 400 s, respectively. The delta Hz was measured for 30 min generating long association and disassociation phases. Kinetic binding curves were used to calculate the apparent affinity (K_D_) and association (k_a_) and disassociation (k_d_) rates for various lipoprotein species. Two independent experiments were conducted using Bruch’s membrane (BrM) from two subjects (N = 2) to support the reproducibility of the findings.

## Supplementary Material

Appendix 01 (PDF)

## Data Availability

Shared data from this publication has been made available through Harvard Dataverse (51). All other data are included in the article and/or *SI Appendix*.
