# Supplementary material for: Bruch’s membrane heparan sulfate retains lipoproteins in the early stages of age-related macular degeneration
Source: Proc Natl Acad Sci U S A. 2025 Jun 13;122(24):e2500727122. doi: 10.1073/pnas.2500727122 (PMC12184332; doi:10.1073/pnas.2500727122)
Supplement: Supplementary file 1 — Appendix 01 (PDF) [file pnas.2500727122.sapp.pdf]

Bruch's Membrane Heparan Sulfate Retains Lipoproteins in the Early Stages of Age-Related Macular Degeneration

Christopher B. Toomey, MD, PhD [1,2]; Savanna Pflugmacher, BS [1]; Kamalu Park [1]; Jessica Pihl PhD [2,3]; Sammy Weiser Novak MS[5]; Jessica Rodriguez, BS [2,3]; Maryam Jalali Ph.D.[1,2], Jaesoo Jung PhD [2,3]; Madeline Mozafari BS [1,2], Sima P. Omran MS[1,2], Cameron K. Pormir[2], Jill Hauer PhD [2,3]; Chelsea Painter PhD [2], Evan Walker [1], Alex S. Huang, MD, PhD [1]; Daniela Boassa PhD [5]; James T. Handa MD [6]; Teodor Aastrup PhD [7]; Philip L.S.M. Gordts, PhD [2,8] and Jeffrey D. Esko, PhD [2,3];

[1] Viterbi Family Department of Ophthalmology and Shiley Eye Institute, University of California San Diego, La Jolla, CA
[2] Glycobiology Research and Training Center, University of California San Diego, La Jolla, CA
[3] Department of Cellular and Molecular Medicine, University of California San Diego, La Jolla, CA
[4] Division of Biomedical Informatics, Department of Medicine, University of California San Diego, La Jolla, CA
[5] Waitt Advanced Biophotonic Core, Salk Institute, La Jolla, CA
[6] Wilmer Eye Institute, Johns Hopkins Medical Institute, Baltimore, MD
[7]Attana AB, Stockholm, Sweden
[8] Department of Medicine, University of California San Diego, La Jolla, CA

Corresponding Author:
Christopher Toomey, MD, PhD
Shiley Eye Institute and Viterbi Family Department of Ophthalmology
University of California San Diego,
9415 Campus Point Dr, MC 0946,
La Jolla, CA 92093-0946, USA
Phone: (858) 361-1344
Email: c1toomey@health.ucsd.edu

This PDF file includes:

Figure S1-3
Table S1-3

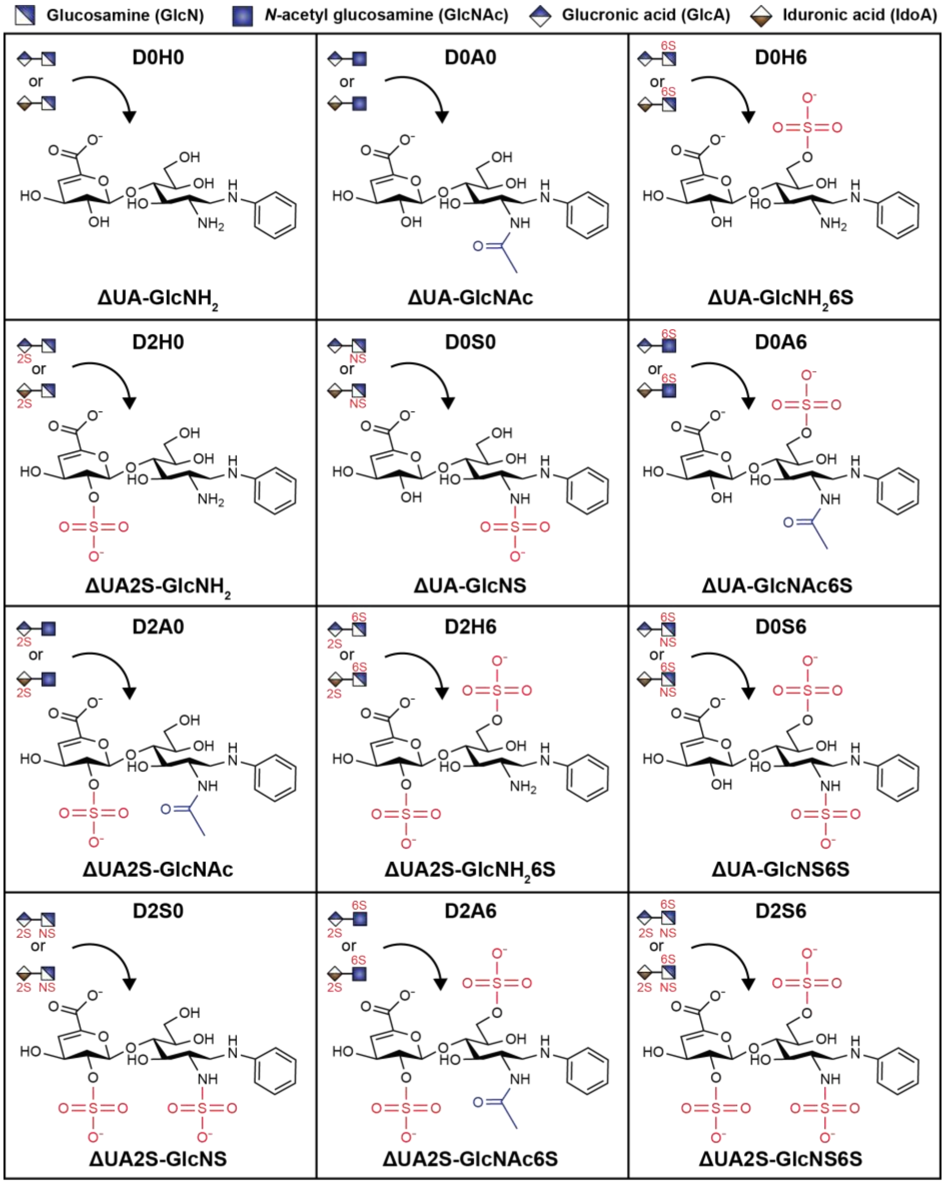

Figure S1: Structures of disaccharide units used for glycosaminoglycan analysis.
HS was isolated from BrM and enzymatically digested using a combination of heparinase I, II, and III to generate specific disaccharide units. The liberated disaccharides were subsequently labeled with aniline to enhance detection. The figure illustrates the resulting structures. The original disaccharide structures are displayed in the top-left corner, with arrows indicating the enzymatic cleavage and aniline-tagging steps. Each disaccharide is annotated with its sulfation patterns and modifications, including 2-O-sulfation (2S), 6-O-sulfation (6S), and N-sulfation (NS), as well as unsulfated residues.

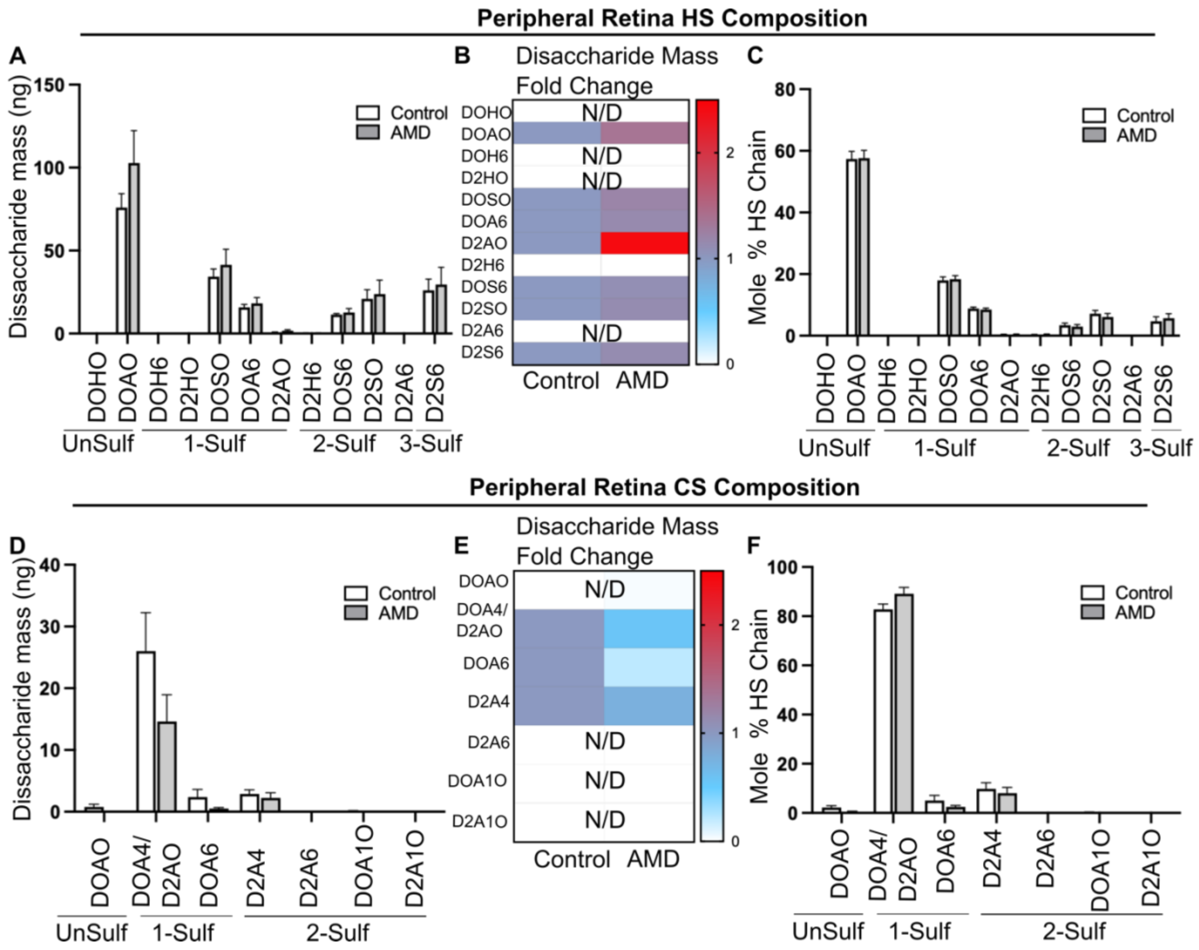

Figure S2: Glycosaminoglycan analysis of AMD peripheral BrM.
(**A-C**) Peripheral BrM HS disaccharide composition analysis shows no significant increase in HS disaccharide content in AMD patients (N=5 subjects, 8 eyes) compared to aged controls (N=7 subjects, 12 eyes). (**E-F**) Peripheral BrM chondroitin sulfate composition was also unchanged.

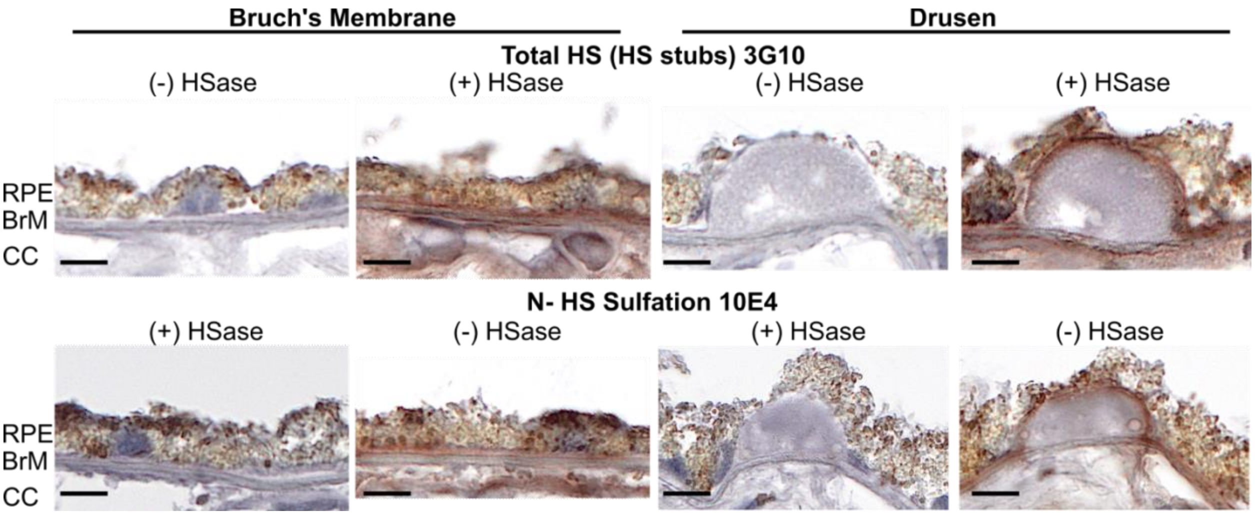

Figure S3: Heparan sulfate is abundant in BrM and underlying drusen.
Representative histology sections from aged controls (86 year-old female without AMD) with BrM (left) and patient with early AMD (90 year-old male) with small drusen (right) stained for heparan sulfate specific antibodies (3G10, top panel; 10E4, bottom panel). HSase treatment creates a unique stub epitope on HS recognized by 3G10 antibody revealing the presence of total heparan sulfate chains, which are rich in normal BrM (left, top panel) and surround drusen in AMD (right, top panel). 10E4 antibody recognizes N-sulfated HS epitope and is removed with HSase treatment as a control (bottom panel). mAb 10E4 stains BrM in aged healthy controls (left, bottom panel) and material overlying drusen in AMD. Staining is notably absent underlying drusen (right, bottom panel). RPE – retinal pigmented epithelium, BrM – Bruch's membrane, CC – choriocapillaris. Scale bar – 10 μm.

Table S1: Bruch's Membrane Heparan Sulfate Composition in AMD

	Control<br>n = 11 subjects; 17 eyes	AMD<br>n = 7 subjects; 12 eyes	p-value
Age	76.5 (70.8, 82.3)	81.3 (70.9, 91.7)	0.326
Sex<br>Female<br>Male	 3 (27.3%)<br>8 (72.7%)	 3 (42.9%)<br>4 (57.1%)	 0.627
**Total HS (ng)**	**335 (225, 445)**	**528 (391, 665)**	**0.047**
Total Protein (μg)	162 (86.5, 237)	150 (56.3, 244)	0.856
D0H0 (ng)	0.08 (-0.09, 0.25)	0.15 (-0.05, 0.35)	0.587
**D0A0** (ng)	**174 (123, 225)**	**270 (206, 334)**	**0.035**
D0H6 (ng)	0.00 (-0.05, 0.05)	0.07 (0.01, 0.12)	0.087
D2H0 (ng)	0 (-, -)	0 (-, -)	-
D0S0 (ng)	62.6 (36.7, 88.5)	100.4 (68.1, 132.7)	0.092
D0A6 (ng)	32.9 (23.2, 42.6)	48.3 (36.2, 60.4)	0.070
D2A0 (ng)	1.24 (-0.28, 2.77)	3.07 (1.17, 4.97)	0.162
D2H6 (ng)	0.71 (-1.26, 2.68)	2.21 (-0.26, 4.67)	0.367
D0S6 (ng)	13.4 (6.89, 19.9)	19.6 (11.5, 27.6)	0.259
D2S0 (ng)	29.1 (13.9, 44.2)	42.9 (23.9, 61.8)	0.281
D2A6 (ng)	0.01 (-0.06, 0.08)	0.07 (-0.02, 0.16)	0.324
D2S6 (ng)	21.0 (7.46, 34.5)	41.0 (24.2, 57.8)	0.087

Table S2: Bruch's Membrane Heparan Sulfate Composition between Chromosome 10q26 Genotypes

	Chr 10, G:G n = 10 subjects; 17 eyes	Chr 10, A:G n = 7 subjects; 11 eyes	p-value
Age	81.7 (74.1, 89.3)	73.1 (67.0, 79.2)	0.079
Sex			
Female	2 (20.0%)	3 (42.9%)	0.593
Male	8 (80.0%)	4 (57.1%)	
Total HS (ng)	449 (319, 579)	377 (221, 532)	0.493
Total Protein (μg)	187 (110, 265)	129 (36.8, 222)	0.361
D0H0 (ng)	0.11 (-0.06, 0.29)	0.11 (-0.10, 0.32)	0.979
D0A0 (ng)	239 (180, 298)	185 (114, 256)	0.266
D0H6 (ng)	0.05 (-0.00, 0.10)	0.00 (-0.06, 0.06)	0.255
D2H0 (ng)	0 (-, -)	0 (-, -)	0 (-, -)
D0S0 (ng)	85.5 (55.9, 115.0)	70.9 (35.5, 106.4)	0.545
D0A6 (ng)	44.0 (33.1, 54.8)	33.8 (20.8, 46.8)	0.258
D2A0 (ng)	2.3 (0.5, 4.0)	1.5 (-0.6, 3.6)	0.597
D2H6 (ng)	0.6 (-1.5, 2.6)	2.0 (-0.5, 4.5)	0.399
D0S6 (ng)	13.8 (6.8, 20.8)	19.0 (10.6, 27.5)	0.367
D2S0 (ng)	36.8 (20.0, 53.5)	33.2 (13.1, 53.3)	0.794
D2A6 (ng)	0.06 (-0.01, 0.14)	0.00 (-0.09, 0.09)	0.314
D2S6 (ng)	27.5 (11.7, 43.3)	31.1 (12.1, 50.1)	0.780

Table S3. Bruch's Membrane Heparan Sulfate Composition between Chromosome 1q32 Genotypes

	Chr 1, T:T<br>n = 9 subjects; 15 eyes	Chr 1, C:T<br>n = 7 subjects; 11 eyes	Chr 1, C:C<br>n = 2 subjects; 3 eyes	p-value
Age	75.8 (66.6, 85.0)	80.6 (73.9, 87.3)	82.5 (76.1, 88.9)	0.532
Sex				
Female	2 (22.2%)	3 (42.9%)	1 (50.0%)	0.509
Male	7 (77.8%)	4 (57.1%)	1 (50.0%)	
Total HS (ng)	376 (244, 509)	490 (339, 640)	285 (3, 568)	0.378
Total Protein (ug)	170 (87, 253)	165 (71, 259)	72 (-105, 249)	0.613
D0H0 (ng)	0.05 (-0.13, 0.22)	0.22 (0.02, 0.42)	0.00 (-0.38, 0.38)	0.384
D0A0 (ng)	192 (130, 255)	251 (180, 322)	160 (26, 292)	0.370
D0H6 (ng)	0.00 (-0.05, 0.05)	0.03 (-0.02, 0.09)	0.14 (0.03, 0.25)	0.088
D2H0 (ng)	0 (-, -)	0 (-, -)	0 (-, -)	-
D0S0 (ng)	69.2 (39.1, 99.3)	95.9 (61.7, 130.1)	49.6 (-14.5, 113.8)	0.369
D0A6 (ng)	35.9 (24.2, 47.7)	44.7 (31.3, 58.1)	32.3 (7.2, 57.3)	0.551
D2A0 (ng)	1.6 (-0.2, 3.5)	2.6 (0.5, 4.6)	1.3 (-2.6, 5.2)	0.757
D2H6 (ng)	2.1 (-0.1, 4.2)	0.03 (-2.5, 2.5)	2.3 (-2.3,7.0)	0.460
D0S6 (ng)	13.1 (6.3, 19.8)	21.7 (14.0, 29.4)	7.7 (-6.8, 22.1)	0.164
D2S0 (ng)	33.7 (16.5, 50.8)	40.5 (21.0, 60.0)	16.8 (-19.7, 53.4)	0.545
D2A6 (ng)	0.01 (-0.07, 0.10)	0.07 (-0.02, 0.16)	0.00 (-0.17, 0.17)	0.610
D2S6 (ng)	27.6 (11.4, 43.9)	34.2 (15.8, 52.7)	15.6 (-19.1, 50.2)	0.642
